# Author Correction: Infectivity, susceptibility, and risk factors associated with SARS-CoV-2 transmission under intensive contact tracing in Hunan, China

**DOI:** 10.1038/s41467-021-22487-4

**Published:** 2021-04-08

**Authors:** Shixiong Hu, Wei Wang, Yan Wang, Maria Litvinova, Kaiwei Luo, Lingshuang Ren, Qianlai Sun, Xinghui Chen, Ge Zeng, Jing Li, Lu Liang, Zhihong Deng, Wen Zheng, Mei Li, Hao Yang, Jinxin Guo, Kai Wang, Xinhua Chen, Ziyan Liu, Han Yan, Huilin Shi, Zhiyuan Chen, Yonghong Zhou, Kaiyuan Sun, Alessandro Vespignani, Cécile Viboud, Lidong Gao, Marco Ajelli, Hongjie Yu

**Affiliations:** 1 Hunan Workstation for Emerging Infectious Disease Control and Prevention, Chinese Academy of Medical Sciences, Hunan Provincial Center for Disease Control and Prevention, Changsha, China; 2 School of Public Health, Fudan University, Key Laboratory of Public Health Safety, Ministry of Education, Shanghai, China; 3 Department of Epidemiology and Biostatistics, Indiana University School of Public Health, Bloomington, IN USA; 4 ISI Foundation, Turin, Italy; 5 West China School of Public Health and West China Fourth Hospital, Sichuan University, Chengdu, Sichuan China; 6 Division of International Epidemiology and Population Studies, Fogarty International Center, National Institutes of Health, Bethesda, MD USA; 7 Laboratory for the Modeling of Biological and Socio-technical Systems, Northeastern University, Boston, MA USA; 8 Department of Infectious Diseases, Huashan Hospital, Fudan University, Shanghai, China; 9 Shanghai Key Laboratory of Infectious Diseases and Biosafety Emergency Response, Shanghai, China; 10 Shanghai Institute of Infectious Disease and Biosecurity, Fudan University, Shanghai, China

**Keywords:** SARS-CoV-2, Viral epidemiology, Preventive medicine, Epidemiology

Correction to: *Nature Communications* 10.1038/s41467-021-21710-6, published online 9 March 2021.

The original version of this Article contained an error in the first sentence of the introduction, which incorrectly read ‘The outbreak of coronavirus disease 2019 (COVID-19) was first detected in December 2009 in Wuhan, China’. The first sentence of the introduction has been corrected to ‘The outbreak of coronavirus disease 2019 (COVID-19) was first detected in December 2019 in Wuhan, China’ in both the PDF and HTML versions of the Article.

